# Supplementary material for: A resource for improved predictions of Trypanosoma and Leishmania protein three-dimensional structure
Source: PLoS One. 2021 Nov 11;16(11):e0259871. doi: 10.1371/journal.pone.0259871 (PMC8584756; doi:10.1371/journal.pone.0259871)
Supplement: S1 Table — (DOCX) [file pone.0259871.s001.docx]

S1 Table. Genome and transcriptome data used to generate the Discoba protein database.

| **type** | **database** | **predicted transcriptome name** | **database accession** | **bioproject accession** | **protein sequence gather strategy** | **reference** |
| --- | --- | --- | --- | --- | --- | --- |
| genome | NCBI SRA | Porcisia_deanei | SRR1662197 | PRJNA267749 | velvet genome assembly and orfs >=100bp | [58] |
| genome | NCBI SRA | Leishmania_hertigi | SRR1662199 | PRJNA267749 | velvet genome assembly and orfs >=100bp | [58] |
| genome | NCBI SRA | Leishmania_pifanoi | SRR1662200 | PRJNA267749 | velvet genome assembly and orfs >=100bp | [58] |
| genome | NCBI SRA | Endotrypanum_schaudinni | SRR1662202 | PRJNA267749 | velvet genome assembly and orfs >=100bp | [58] |
| genome | NCBI SRA | Leishmania_naiffi | SRR1657911 | PRJNA267749 | velvet genome assembly and orfs >=100bp | [58] |
| genome | NCBI SRA | Leishmania_sp_LTCP19748 | SRR1185708 | PRJNA157075 | velvet genome assembly and orfs >=100bp |  |
| genome | NCBI SRA | Kinetoplastid_LTCP393 | SRR5973234 | PRJNA398352 | velvet genome assembly and orfs >=100bp | [59] |
| genome | NCBI SRA | Kinetoplastid_LH21 | SRR5973232 | PRJNA398352 | velvet genome assembly and orfs >=100bp | [59] |
| genome | NCBI SRA | Kinetoplastid_LTCP15171 | SRR5973231 | PRJNA398352 | velvet genome assembly and orfs >=100bp | [59] |
| genome | NCBI SRA | Kinetoplastid_LVH60 | SRR5973230 | PRJNA398352 | velvet genome assembly and orfs >=100bp | [59] |
| genome | NCBI SRA | Kinetoplastid_LH23 | SRR5973229 | PRJNA398352 | velvet genome assembly and orfs >=100bp | [59] |
| genome | NCBI SRA | Kinetoplastid_LVH60a | SRR5973228 | PRJNA398352 | velvet genome assembly and orfs >=100bp | [59] |
| genome | NCBI SRA | Kinetoplastid_HUUFS14 | SRR5973233 | PRJNA398352 | velvet genome assembly and orfs >=100bp | [59] |
| genome | NCBI SRA | Trypanosomatidae_sp_Fi-14 | SRR9090237 | PRJNA543408 | velvet genome assembly and orfs >=100bp | [60] |
| genome | NCBI SRA | Trypanosoma_caninum | SRR8923841 | PRJNA532385 | velvet genome assembly and orfs >=100bp |  |
| genome | NCBI SRA | Trypanosoma_cruzi_ATCC_30160 | SRR2057744, SRR2057750, SRR2057802 | PRJNA284532 | velvet genome assembly and orfs >=100bp |  |
| genome | NCBI SRA | Trypanosoma_cruzi_TRYCC_1522 | SRR2057752, SRR2057745, SRR2057791, SRR2057774 | PRJNA284534 | velvet genome assembly and orfs >=100bp |  |
| single cell genome | NCBI SRA | Diplonemid_21sb | SRR4022277 | PRJNA338474 | velvet genome assembly and orfs >=100bp | [61] |
| single cell genome | NCBI SRA | Diplonemid_9sb | SRR4022276 | PRJNA338474 | velvet genome assembly and orfs >=100bp | [61] |
| single cell genome | NCBI SRA | Diplonemid_4sb | SRR4022275 | PRJNA338474 | velvet genome assembly and orfs >=100bp | [61] |
| single cell genome | NCBI SRA | Diplonemid_1sb | SRR4022274 | PRJNA338474 | velvet genome assembly and orfs >=100bp | [61] |
| single cell genome | NCBI SRA | Diplonemid_47 | SRR4022273 | PRJNA338474 | velvet genome assembly and orfs >=100bp | [61] |
| single cell genome | NCBI SRA | Diplonemid_37 | SRR4022272 | PRJNA338474 | velvet genome assembly and orfs >=100bp | [61] |
| single cell genome | NCBI SRA | Diplonemid_27 | SRR4022272 | PRJNA338474 | velvet genome assembly and orfs >=100bp | [61] |
| single cell genome | NCBI SRA | Diplonemid_21 | SRR4022272 | PRJNA338474 | velvet genome assembly and orfs >=100bp | [61] |
| single cell genome | NCBI SRA | Diplonemid_13 | SRR4022272 | PRJNA338474 | velvet genome assembly and orfs >=100bp | [61] |
| single cell genome | NCBI SRA | Diplonemid_3 | SRR4022268 | PRJNA338474 | velvet genome assembly and orfs >=100bp | [61] |
| single cell genome | NCBI SRA | Eutreptiella_87 | SRR10442434 | PRJNA379597 | velvet genome assembly and orfs >=100bp | [62,63] |
| single cell genome | NCBI SRA | Kinetoplastida_80 | SRR10442435 | PRJNA379597 | velvet genome assembly and orfs >=100bp | [62,63] |
| single cell genome | NCBI SRA | Diplonemid_94a | SRR10442437 | PRJNA379597 | velvet genome assembly and orfs >=100bp | [62,63] |
| single cell genome | NCBI SRA | Euglenozoa_98 | SRR10442438 | PRJNA379597 | velvet genome assembly and orfs >=100bp | [62,63] |
| single cell genome | NCBI SRA | Diplonema_82 | SRR10442439 | PRJNA379597 | velvet genome assembly and orfs >=100bp | [62,63] |
| single cell genome | NCBI SRA | Euglenida_90 | SRR10442440 | PRJNA379597 | velvet genome assembly and orfs >=100bp | [62,63] |
| single cell genome | NCBI SRA | Kinetoplastida_57 | SRR10442441 | PRJNA379597 | velvet genome assembly and orfs >=100bp | [62,63] |
| single cell genome | NCBI SRA | Euglenozoa_Mix_87 | SRR10442446 | PRJNA379597 | velvet genome assembly and orfs >=100bp | [62,63] |
| single cell genome | NCBI SRA | Hemistasia_100 | SRR10442448 | PRJNA379597 | velvet genome assembly and orfs >=100bp | [62,63] |
| single cell genome | NCBI SRA | Euglenozoa_Bodo_97 | SRR10442451 | PRJNA379597 | velvet genome assembly and orfs >=100bp | [62,63] |
| single cell genome | NCBI SRA | Kinetoplastida_89 | SRR10442453 | PRJNA379597 | velvet genome assembly and orfs >=100bp | [62,63] |
| single cell genome | NCBI SRA | Diplonema_83 | SRR10442455 | PRJNA379597 | velvet genome assembly and orfs >=100bp | [62,63] |
| single cell genome | NCBI SRA | Diplonemid_81 | SRR10442456 | PRJNA379597 | velvet genome assembly and orfs >=100bp | [62,63] |
| single cell genome | NCBI SRA | Neobodo_93 | SRR10442459 | PRJNA379597 | velvet genome assembly and orfs >=100bp | [62,63] |
| single cell genome | NCBI SRA | Diplonemid_96 | SRR10442462 | PRJNA379597 | velvet genome assembly and orfs >=100bp | [62,63] |
| single cell genome | NCBI SRA | Diplonemid_94c | SRR10442466 | PRJNA379597 | velvet genome assembly and orfs >=100bp | [62,63] |
| single cell genome | NCBI SRA | Diplonemid_94b | SRR10442467 | PRJNA379597 | velvet genome assembly and orfs >=100bp | [62,63] |
| single cell genome | NCBI SRA | Kinetoplastida_95 | SRR10442468 | PRJNA379597 | velvet genome assembly and orfs >=100bp | [62,63] |
| single cell genome | NCBI SRA | Apusozoa1 | SRR10442469 | PRJNA379597 | velvet genome assembly and orfs >=100bp | [62,63] |
| single cell genome | NCBI SRA | Diplonemida_93 | SRR10442470 | PRJNA379597 | velvet genome assembly and orfs >=100bp | [62,63] |
| single cell genome | NCBI SRA | Diplonemid_93 | SRR10442472 | PRJNA379597 | velvet genome assembly and orfs >=100bp | [62,63] |
| single cell genome | NCBI SRA | 4B_35A_extra | SRR10499212 | PRJNA379597 | velvet genome assembly and orfs >=100bp | [62,63] |
| genome | NCBI genome | Novymonas_esmeraldas | GCA_019188245.1_ASM1918824v1 | PRJNA681813 | orfs >=100bp | [64] |
| genome | NCBI genome | Phytomonas_serpens_9T | GCA_000331125.1_PhytSerpensv01 | PRJNA80957 | orfs >=100bp | [65] |
| genome | NCBI genome | Angomonas_desouzai | GCA_000482185.1_Ades_1.0 | PRJNA203515 | orfs >=100bp | [66] |
| genome | NCBI genome | Strigomonas_culicis | GCA_000482145.1_Scul_1.0 | PRJNA203517 | orfs >=100bp | [66] |
| genome | NCBI genome | Strigomonas_galati | GCA_000482125.1_Sgal_1.0 | PRJNA203516 | orfs >=100bp | [66] |
| genome | NCBI genome | Strigomonas_oncopelti | GCA_000482165.1_Sonc_1.0 | PRJNA203518 | orfs >=100bp | [66] |
| genome | NCBI genome | Crithidia_acanthocephali | GCA_000482105.1_Caca_1.0 | PRJNA203520 | orfs >=100bp | [66] |
| genome | NCBI genome | Crithidia_bombi | GCA_900240985.1_crithidia-bombi.GDC.2013.v1 | PRJEB21108 | orfs >=100bp | [67] |
| genome | NCBI genome | Crithidia_expoeki | GCA_900240875.1_crithidia-expoeki.GDC.2015.v1 | PRJEB21109 | orfs >=100bp | [67] |
| genome | NCBI genome | Crithidia_mellificae | GCA_002216565.1_ASM221656v1 | PRJNA319526 | orfs >=100bp | [68] |
| genome | NCBI genome | Leishmania_sp_AIIMS-LM-SS-PKDL-LD-974 | GCA_000981925.2_Ld_v2 | PRJNA280137 | orfs >=100bp | [69] |
| genome | NCBI genome | Lotmaria_passim | GCA_000635995.1_ASM63599v1 | PRJNA78249 | orfs >=100bp | [68] |
| genome | NCBI genome | Herpetomonas_muscarum | GCA_000482205.1_Hmus_1.0 | PRJNA203519 | orfs >=100bp | [66] |
| genome | NCBI genome | Leishmania_chagasi | GCA_014466975.1_ASM1446697v1 | PRJNA523126 | orfs >=100bp |  |
| genome | NCBI genome | Leishmania_guyanensis | GCA_003664525.1_ASM366452v1 | PRJNA484340 | orfs >=100bp | [70] |
| genome | NCBI genome | Leishmania_lainsoni | GCA_003664395.1_CDC_Llain_216-34_v1 | PRJNA484340 | orfs >=100bp | [71] |
| genome | NCBI genome | Endotrypanum_monterogeii | GCA_000333855.2_Endotrypanum_monterogeii-LV88-1.0.3 | PRJNA165953 | orfs >=100bp | [72] |
| genome | TriTrypDB genome | Trypanosoma_cruzi_Ycl6 | TcruziYcl6 |  | orfs >=100bp | [73] |
| genome | TriTrypDB genome | Trypanosoma_cruzi_Ycl4 | TcruziYcl4 |  | orfs >=100bp | [73] |
| genome | TriTrypDB genome | Trypanosoma_cruzi_Ycl2 | TcruziYcl2 |  | orfs >=100bp | [73] |
| genome | TriTrypDB genome | Trypanosoma_cruzi_Y | TcruziY |  | orfs >=100bp | [74] |
| genome | TriTrypDB genome | Trypanosoma_cruzi_S92a | TcruziS92a |  | orfs >=100bp | [73] |
| genome | TriTrypDB genome | Trypanosoma_cruzi_S44a | TcruziS44a |  | orfs >=100bp | [73] |
| genome | TriTrypDB genome | Trypanosoma_cruzi_S23b | TcruziS23b |  | orfs >=100bp | [73] |
| genome | TriTrypDB genome | Trypanosoma_cruzi_S162a | TcruziS162a |  | orfs >=100bp | [73] |
| genome | TriTrypDB genome | Trypanosoma_cruzi_S154a | TcruziS154a |  | orfs >=100bp | [73] |
| genome | TriTrypDB genome | Trypanosoma_cruzi_S15 | TcruziS15 |  | orfs >=100bp | [73] |
| genome | TriTrypDB genome | Trypanosoma_cruzi_S11 | TcruziS11 |  | orfs >=100bp | [73] |
| genome | TriTrypDB genome | Trypanosoma_cruzi_231 | Tcruzi231 |  | orfs >=100bp | [75,76] |
| genome | TriTrypDB genome | Trypanosoma_cruzi_Bug2148 | TcruziBug2148 |  | orfs >=100bp | [77] |
| genome | TriTrypDB genome | Trypanosoma_cruzi_Esmeraldo | TcruziEsmeraldo |  | orfs >=100bp | Gregory A. Buck |
| genome | TriTrypDB genome | Trypanosoma_cruzi_JRcl4 | TcruziJRcl4 |  | orfs >=100bp | Wes Warren |
| genome | TriTrypDB genome | Trypanosoma_cruzi_Tulacl2 | TcruziTulacl2 |  | orfs >=100bp | [78] |
| genome | TriTrypDB genome | Leishmania_donovani_BHU1220 | LdonovaniBHU1220 | PRJNA192928 | orfs >=100bp | [79] |
| transcriptome | NCBI SRA | Prokinetoplastina_PhM-4 | SRR9613186 | PRJNA549754 | trinity transcriptome assembly and transdecoder | [80] |
| transcriptome | NCBI SRA | Prokinetoplastina_PhF-6 | SRR9613187 | PRJNA549754 | trinity transcriptome assembly and transdecoder | [80] |
| transcriptome | NCBI SRA | Rhynchopus_humris | SRR9588121, SRR9588122, SRR9588122 | PRJNA550027 | trinity transcriptome assembly and transdecoder | [7] |
| transcriptome | NCBI SRA | Sulcionema_specki | SRR9334250, SRR9334251 | PRJNA550027 | trinity transcriptome assembly and transdecoder | [7] |
| transcriptome | NCBI SRA | Azumiobodo_hoyamushi | SRR10586159 | PRJNA344936 | trinity transcriptome assembly and transdecoder | [81] |
| transcriptome | NCBI SRA | Namystynia_karyoxenos | SRR8676451 | PRJNA525750 | trinity transcriptome assembly and transdecoder | [82] |
| transcriptome | NCBI SRA | Lacrimia_lanifica | SRR8676452 | PRJNA525750 | trinity transcriptome assembly and transdecoder | [82] |
| transcriptome | NCBI SRA | Diplonema_japonicum | SRR8676453 | PRJNA525750 | trinity transcriptome assembly and transdecoder | [82] |
| transcriptome | NCBI SRA | Artemidia_motanka | SRR8676455 | PRJNA525750 | trinity transcriptome assembly and transdecoder | [82] |
| transcriptome | NCBI SRA | Diplonema_sp.2 | SRR5998375 | PRJNA392339 | trinity transcriptome assembly and transdecoder | [83] |
| transcriptome | NCBI SRA | Diplonema_ambulator | SRR5998378, SRR5998379 | PRJNA392339 | trinity transcriptome assembly and transdecoder | [83] |
| transcriptome | NCBI SRA | Rhynchopus_euleeides | SRR5998383 | PRJNA392339 | trinity transcriptome assembly and transdecoder | [83] |
| transcriptome | NCBI SRA | Willaertia_magna | ERR3764909 | PRJEB30797 | trinity transcriptome assembly and transdecoder | [84] |
| transcriptome | NCBI SRA | Phytomonas_francai | ERR1655129, ERR1655128 | PRJEB15491 | trinity transcriptome assembly and transdecoder | [85] |
| transcriptome | NCBI SRA | Vickermania_ingenoplastis_CP021 | SRR13015660 | PRJNA675748 | trinity transcriptome assembly and transdecoder | [86] |
| transcriptome | NCBI SRA | Trypanosoma_carassii | SRR5120186 | PRJNA358054 | trinity transcriptome assembly and transdecoder | [87] |
| transcriptome | NCBI SRA | Porcisia_deanei | SRR13125062 | PRJNA680237 | trinity transcriptome assembly and transdecoder | [88] |
| transcriptome | NCBI SRA | Crithidia_thermophila | SRR11278472, SRR11278473, SRR11278474 | PRJNA611063 | trinity transcriptome assembly and transdecoder | [89] |
| transcriptome | NCBI SRA | Andalucia_incarcerata | SRR2566811 | PRJNA297797 | trinity transcriptome assembly and transdecoder | [90] |
| transcriptome | NCBI SRA | Neovahlkampfia_damariscottae | SRR9328295, SRR9328296 | PRJNA549687 | trinity transcriptome assembly and transdecoder | [91] |
| transcriptome | NCBI SRA | Ankaliazontas_spiralis_PhF-5__Parabodo_caudatus | SRR13394430 | PRJNA549754 | trinity transcriptome assembly and transdecoder | [80] |
| transcriptome | NCBI TSA | Hemistasia_phaeocysticola | GHOA | PRJNA549599 | transdecoder | [7] |
| transcriptome | NCBI TSA | Trypanoplasma_borreli_Tt-JH | GHOB | PRJNA549827 | transdecoder | [7] |
| transcriptome | NCBI TSA | Rhabdomonas_costata | GJGC | PRJNA550357 | transdecoder | [92] |
| transcriptome | NCBI TSA | Trypanoplasma_borreli | GFCF | PRJNA354696 | transdecoder | [93] |
| transcriptome | NCBI TSA | Euglena_gracilis | GDJR | PRJNA289402 | transdecoder | [94] |
| transcriptome | NCBI TSA | Euglena_longa | GGOE | PRJNA471257 | transdecoder | [95] |
| transcriptome | NCBI TSA | Pharyngomonas_kirbyi | GECH | PRJNA301448 | transdecoder | [96] |
| transcriptome | NCBI TSA | Percolomonas_cosmopolitus | HBGD | PRJEB37117 | transdecoder | [97] |
| transcriptome | NCBI TSA | Euglena_gracilis_2 | GEFR | PRJNA298469 | transdecoder | [98] |
| transcriptome | NCBI TSA | Trypanoplasma_borreli_DieterSteinhagen | GFCF | PRJNA354696 | transdecoder | [93] |
| transcriptome | NCBI TSA | Percolomonas_cosmopolitus_WS | HBGD | PRJEB37117 | transdecoder | [97] |
| transcriptome | MMETSP | Neobodo_designis | MMETSP1114 | PRJNA248394 | provided by MMETSP (transdecoder) | [97] |
| transcriptome | MMETSP | Eutreptiella_gymnastica | MMETSP0039 | PRJNA248394 | provided by MMETSP (transdecoder) | [97] |
| transcriptome | NCBI genome | Bodo_saltans | GCA_001460835.1_BSAL | PRJEB10421 | provided by NCBI | [99] |
| transcriptome | NCBI genome | Perkinsela_sp | GCA_001235845.1_ASM123584v1 | PRJNA194468 | provided by NCBI | [100] |
| transcriptome | NCBI genome | Naegleria_fowleri | GCA_008403515.1_ASM840351v1 | PRJNA541227 | provided by NCBI | [101] |
| transcriptome | NCBI genome | Naegleria_gruberi | GCF_000004985.1_V1.0 | PRJNA14010 | provided by NCBI | [102] |
| transcriptome | NCBI genome | Andalucia_godoyi | GCA_009859145.1_Andalucia_godoyi_V16 | PRJNA559352 | provided by NCBI | [103] |
| transcriptome | NCBI genome | Naegleria_lovaniensis | GCA_003324165.2_Nlova_2.1 | PRJNA445795 | provided by NCBI | [104] |
| transcriptome | NCBI genome | Perkinsela_sp_CCAP-1560-4 | GCA_001235845.1_ASM123584v1 | PRJNA194468 | provided by NCBI | [100] |
| transcriptome | NCBI genome | Trypanosoma_conorhini | GCF_003719485.1_ASM371948v1 | PRJNA315397 | provided by NCBI | [105] |
| transcriptome | NCBI genome | Phytomonas_sp_isolate_EM1 | GCA_000582765.1_AKH_PRJEB1535_v1 | PRJEB1535 | provided by NCBI | [106] |
| transcriptome | NCBI genome | Trypanosoma_equiperdum | GCA_001457755.2_Trypanosoma_equiperdum_OVI_V2 | PRJEB11407 | provided by NCBI | [107] |
| transcriptome | NCBI genome | Leishmania_orientalis | GCA_017916335.1_LU_Lori_1.0 | PRJNA691532 | provided by NCBI | [108] |
| transcriptome | NCBI genome | Leishmania_martiniquensis | GCA_017916325.1_LU_Lmar_1.0 | PRJNA691531 | provided by NCBI | [109] |
| transcriptome | NCBI genome | Leishmania_enriettii | GCA_017916305.1_LU_Lenr_1.0 | PRJNA691534 | provided by NCBI | [58] |
| transcriptome | NCBI genome | Porcisia_hertigi | GCA_017918235.1_LU_Pher_1.0 | PRJNA691541 | provided by NCBI | [88] |
| transcriptome | TriTrypDB genome | Trypanosoma_vivax_Y486 | TvivaxY486 |  | provided by TriTrypDB | GeneDB |
| transcriptome | TriTrypDB genome | Trypanosoma_theileri_Edinburgh | TtheileriEdinburgh |  | provided by TriTrypDB | [110,111] |
| transcriptome | TriTrypDB genome | Trypanosoma_rangeli_SC58 | TrangeliSC58 |  | provided by TriTrypDB | [112] |
| transcriptome | TriTrypDB genome | Trypanosoma_grayi_ANR4 | TgrayiANR4 |  | provided by TriTrypDB | [87] |
| transcriptome | TriTrypDB genome | Trypanosoma_evansi_STIB805 | TevansiSTIB805 |  | provided by TriTrypDB | [113] |
| transcriptome | TriTrypDB genome | Trypanosoma_cruzi_YC6 | TcruziYC6 |  | provided by TriTrypDB | [114] |
| transcriptome | TriTrypDB genome | Trypanosoma_cruzi_TCC | TcruziTCC |  | provided by TriTrypDB | [115] |
| transcriptome | TriTrypDB genome | Trypanosoma_cruzi_SylvioX10-1 | TcruziSylvioX10-1 |  | provided by TriTrypDB | [116,117] |
| transcriptome | TriTrypDB genome | Trypanosoma_cruzi_SylvioX10-1-2012 | TcruziSylvioX10-1-2012 |  | provided by TriTrypDB | [116,117] |
| transcriptome | TriTrypDB genome | Trypanosoma_cruzi_MarinkelleiB7 | TcruzimarinkelleiB7 |  | provided by TriTrypDB | [117] |
| transcriptome | TriTrypDB genome | Trypanosoma_cruzi_Dm28c-2018 | TcruziDm28c2018 |  | provided by TriTrypDB | [115,118] |
| transcriptome | TriTrypDB genome | Trypanosoma_cruzi_Dm28c-2017 | TcruziDm28c2017 | PRJNA330977 | provided by TriTrypDB | Christian Probst |
| transcriptome | TriTrypDB genome | Trypanosoma_cruzi_Dm28c-2014 | TcruziDm28c2014 |  | provided by TriTrypDB | [119] |
| transcriptome | TriTrypDB genome | Trypanosoma_cruzi_Brener_nonEL | TcruziCLBrenerNon-Esmeraldo-like |  | provided by TriTrypDB | [120,121] |
| transcriptome | TriTrypDB genome | Trypanosoma_cruzi_Brener_EL | TcruziCLBrenerEsmeraldo-like |  | provided by TriTrypDB | [120,121] |
| transcriptome | TriTrypDB genome | Trypanosoma_cruzi_Brener | TcruziCLBrener |  | provided by TriTrypDB | [120,121] |
| transcriptome | TriTrypDB genome | Trypanosoma_cruzi_BrazilA4 | TcruziBrazilA4 |  | provided by TriTrypDB | [114] |
| transcriptome | TriTrypDB genome | Trypanosoma_congolense_IL3000-2019 | TcongolenseIL3000_2019 |  | provided by TriTrypDB | [122] |
| transcriptome | TriTrypDB genome | Trypanosoma_congolense_IL3000 | TcongolenseIL3000 |  | provided by TriTrypDB | [123] |
| transcriptome | TriTrypDB genome | Trypanosoma_brucei_TREU927 | TbruceiTREU927 |  | provided by TriTrypDB | [124] |
| transcriptome | TriTrypDB genome | Trypanosoma_brucei_Lister427-2018 | TbruceiLister427_2018 |  | provided by TriTrypDB | [125] |
| transcriptome | TriTrypDB genome | Trypanosoma_gambiense_DAL972 | TbruceigambienseDAL972 |  | provided by TriTrypDB | [126] |
| transcriptome | TriTrypDB genome | Paratrypanosoma_confusum_CUL13 | PconfusumCUL13 |  | provided by TriTrypDB | [127] |
| transcriptome | TriTrypDB genome | Leishmania_turanica_LEM423 | LturanicaLEM423 |  | provided by TriTrypDB | [72] |
| transcriptome | TriTrypDB genome | Leishmania_tropica_L590 | LtropicaL590 |  | provided by TriTrypDB | [128] |
| transcriptome | TriTrypDB genome | Leishmania_tarentolae_ParrotTarlII | LtarentolaeParrotTarII |  | provided by TriTrypDB | [129] |
| transcriptome | TriTrypDB genome | Leishmania_sp_MARLEM2494 | LspMARLEM2494 |  | provided by TriTrypDB | [72] |
| transcriptome | TriTrypDB genome | Leptomonas_seymouri_ATCC30220 | LseymouriATCC30220 |  | provided by TriTrypDB | [130] |
| transcriptome | TriTrypDB genome | Leptomonas_pyrrhocoris_H10 | LpyrrhocorisH10 |  | provided by TriTrypDB | [131] |
| transcriptome | TriTrypDB genome | Leishmania_panamensis_MHOMPA94PSC1 | LpanamensisMHOMPA94PSC1 |  | provided by TriTrypDB | [132] |
| transcriptome | TriTrypDB genome | Leishmania_panamensis_MHOMCOL81L13 | LpanamensisMHOMCOL81L13 |  | provided by TriTrypDB | [72] |
| transcriptome | TriTrypDB genome | Leishmania_mexicana_MHOMGT2001U1103 | LmexicanaMHOMGT2001U1103 |  | provided by TriTrypDB | [133] |
| transcriptome | TriTrypDB genome | Leishmania_majorSD75-1 | LmajorSD75.1 |  | provided by TriTrypDB | [72] |
| transcriptome | TriTrypDB genome | Leishmania_major_LV39c5 | LmajorLV39c5 |  | provided by TriTrypDB | [72] |
| transcriptome | TriTrypDB genome | Leishmania_major_Friedlin | LmajorFriedlin |  | provided by TriTrypDB | [134] |
| transcriptome | TriTrypDB genome | Leishmania_infantum_JPCM5 | LinfantumJPCM5 |  | provided by TriTrypDB | [134] |
| transcriptome | TriTrypDB genome | Leishmania_gerbilli_LEM452 | LgerbilliLEM452 |  | provided by TriTrypDB | [72] |
| transcriptome | TriTrypDB genome | Leishmania_enriettii_LEM3045 | LenriettiiLEM3045 |  | provided by TriTrypDB | [72] |
| transcriptome | TriTrypDB genome | Leishmania_donovani_CL-SL | LdonovaniCL-SL |  | provided by TriTrypDB | [135,136] |
| transcriptome | TriTrypDB genome | Leishmania_donovani_BPK282A1 | LdonovaniBPK282A1 |  | provided by TriTrypDB | [137] |
| transcriptome | TriTrypDB genome | Leishmania_braziliensis_MHOMBR75M2904-2019 | LbraziliensisMHOMBR75M2904_2019 |  | provided by TriTrypDB | James Cotton |
| transcriptome | TriTrypDB genome | Leishmania_braziliensis_MHOMBR75M2904 | LbraziliensisMHOMBR75M2904 |  | provided by TriTrypDB | [138] |
| transcriptome | TriTrypDB genome | Leishmania_braziliensis_MHOMBR75M2903 | LbraziliensisMHOMBR75M2903 |  | provided by TriTrypDB | [72] |
| transcriptome | TriTrypDB genome | Leishmania_arabica_LEM1108 | LarabicaLEM1108 |  | provided by TriTrypDB | [72] |
| transcriptome | TriTrypDB genome | Leishmania_amazonensis_MHOMBR71973M2269 | LamazonensisMHOMBR71973M2269 |  | provided by TriTrypDB | [139] |
| transcriptome | TriTrypDB genome | Leishmania_aethiopica_L147 | LaethiopicaL147 |  | provided by TriTrypDB | [72] |
| transcriptome | TriTrypDB genome | Endotrypanum_monterogeii_LV88 | EmonterogeiiLV88 |  | provided by TriTrypDB | [72] |
| transcriptome | TriTrypDB genome | Crithidia_fasciculata_CfCl | CfasciculataCfCl |  | provided by TriTrypDB | [72] |
| transcriptome | TriTrypDB genome | Bodo_saltans_LakeKonstanz | BsaltansLakeKonstanz |  | provided by TriTrypDB | [140] |
| transcriptome | TriTrypDB genome | Blechomonas_ayalai_B08-376 | BayalaiB08-376 |  | provided by TriTrypDB | [141] |
| transcriptome | TriTrypDB genome | Angomonas_deanai_CavalhoATCCPRA-265 | AdeanaiCavalhoATCCPRA-265 |  | provided by TriTrypDB | [142] |
| transcriptome | TriTrypDB genome | Trypanosoma_cruzi_G | TcruziG |  | provided by TriTrypDB | [105] |
| transcriptome | TriTrypDB genome | Trypanosoma_cruzi_CL | TcruziCL |  | provided by TriTrypDB | [105] |
| single cell transcriptome | NCBI SRA | Symbiontida_sp_KSa7 | SRR11528868 | PRJNA624171 | trinity transcriptome assembly and transdecoder | [143] |
| single cell transcriptome | NCBI SRA | Symbiontida_sp_HLA12 | SRR11528869 | PRJNA624171 | trinity transcriptome assembly and transdecoder | [143] |
| single cell transcriptome | NCBI SRA | Heteronema_vittatum_CB2 | SRR11528870 | PRJNA624171 | trinity transcriptome assembly and transdecoder | [143] |
| single cell transcriptome | NCBI SRA | Chasmostoma_nieuportense_CB1 | SRR11528871 | PRJNA624171 | trinity transcriptome assembly and transdecoder | [143] |
| single cell transcriptome | NCBI SRA | Ploeotia_sp_CARIB1 | SRR11528872 | PRJNA624171 | trinity transcriptome assembly and transdecoder | [143] |
| single cell transcriptome | NCBI SRA | Urceolus_sp_BLP5 | SRR11528873 | PRJNA624171 | trinity transcriptome assembly and transdecoder | [143] |
| single cell transcriptome | NCBI SRA | Sphenomonas_quadrangularis_AM6 | SRR11528874 | PRJNA624171 | trinity transcriptome assembly and transdecoder | [143] |
| single cell transcriptome | NCBI SRA | Neometanema_parovale_KM051 | SRR11528875 | PRJNA624171 | trinity transcriptome assembly and transdecoder | [143] |
| single cell transcriptome | NCBI SRA | Ploeotia_vitrea_MX-CHA | SRR11528876 | PRJNA624171 | trinity transcriptome assembly and transdecoder | [143] |
| single cell transcriptome | NCBI SRA | Keelungia_sp_KM082 | SRR11528877 | PRJNA624171 | trinity transcriptome assembly and transdecoder | [143] |
| single cell transcriptome | NCBI SRA | Notosolenus_urceolatus_KM049 | SRR11528878 | PRJNA624171 | trinity transcriptome assembly and transdecoder | [143] |
| single cell transcriptome | NCBI SRA | Olkasia_polycarbonata_UB49 | SRR11528879 | PRJNA624171 | trinity transcriptome assembly and transdecoder | [143] |
| single cell transcriptome | NCBI SRA | Heteronema_vittatum_ABIC3 | SRR11528880 | PRJNA624171 | trinity transcriptome assembly and transdecoder | [143] |
| single cell transcriptome | NCBI SRA | Olkasia_polycarbonata_UB45 | SRR11528881 | PRJNA624171 | trinity transcriptome assembly and transdecoder | [143] |
| single cell transcriptome | NCBI SRA | Liburna_glaciale_UB43 | SRR11528882 | PRJNA624171 | trinity transcriptome assembly and transdecoder | [143] |
| single cell transcriptome | NCBI SRA | Liburna_glaciale_UB37 | SRR11528883 | PRJNA624171 | trinity transcriptome assembly and transdecoder | [143] |
| single cell transcriptome | NCBI SRA | Dinema_litorale_UB26 | SRR11528884 | PRJNA624171 | trinity transcriptome assembly and transdecoder | [143] |
| single cell transcriptome | NCBI SRA | Anisonema_acinus_SMS2 | SRR11528885 | PRJNA624171 | trinity transcriptome assembly and transdecoder | [143] |
| single cell transcriptome | NCBI SRA | Anisonema_acinus_SAL5 | SRR11528886 | PRJNA624171 | trinity transcriptome assembly and transdecoder | [143] |
| single cell transcriptome | NCBI SRA | Peranema_trichophorum_PtR | SRR11528887 | PRJNA624171 | trinity transcriptome assembly and transdecoder | [143] |
| single cell transcriptome | NCBI SRA | Jenningsia_sp_PLL12 | SRR11528888 | PRJNA624171 | trinity transcriptome assembly and transdecoder | [143] |
| single cell transcriptome | NCBI SRA | Urceolus_cf_cornutus_PLL10 | SRR11528889 | PRJNA624171 | trinity transcriptome assembly and transdecoder | [143] |
| single cell transcriptome | NCBI SRA | Lentomonas_cf_corrugata_LEN2 | SRR11528890 | PRJNA624171 | trinity transcriptome assembly and transdecoder | [143] |
| single cell transcriptome | NCBI SRA | Jenningsia_fusiforme_ABIC1 | SRR11528891 | PRJNA624171 | trinity transcriptome assembly and transdecoder | [143] |
| single cell transcriptome | NCBI SRA | Dinema_validum_AB2-2 | SRR11528892 | PRJNA624171 | trinity transcriptome assembly and transdecoder | [143] |
| single cell transcriptome | NCBI SRA | Distigma_sp_N6 | SRR10099989 | PRJNA564423 | trinity transcriptome assembly and transdecoder |  |
| single cell transcriptome | NCBI SRA | Distigma_sp_P2 | SRR10099983 | PRJNA564423 | trinity transcriptome assembly and transdecoder |  |
| single cell transcriptome | NCBI SRA | Distigma_sp_P5 | SRR10099980 | PRJNA564423 | trinity transcriptome assembly and transdecoder |  |
| single cell transcriptome | NCBI SRA | Euglenida_sp_18W | SRR10099986 | PRJNA564423 | trinity transcriptome assembly and transdecoder |  |
| single cell transcriptome | NCBI SRA | Euglenida_sp_3W | SRR10100068 | PRJNA564423 | trinity transcriptome assembly and transdecoder |  |
| single cell transcriptome | NCBI SRA | Euglenida_sp_D1 | SRR10100025 | PRJNA564423 | trinity transcriptome assembly and transdecoder |  |
| single cell transcriptome | NCBI SRA | Euglenida_sp_E4 | SRR10100023 | PRJNA564423 | trinity transcriptome assembly and transdecoder |  |
| single cell transcriptome | NCBI SRA | Euglenida_sp_F2 | SRR10100020 | PRJNA564423 | trinity transcriptome assembly and transdecoder |  |
| single cell transcriptome | NCBI SRA | Euglenida_sp_H2 | SRR10100012 | PRJNA564423 | trinity transcriptome assembly and transdecoder |  |
| single cell transcriptome | NCBI SRA | Euglenida_sp_L1 | SRR10100003 | PRJNA564423 | trinity transcriptome assembly and transdecoder |  |
| single cell transcriptome | NCBI SRA | Euglenida_sp_L5 | SRR10100000 | PRJNA564423 | trinity transcriptome assembly and transdecoder |  |
| single cell transcriptome | NCBI SRA | Euglenida_sp_N4 | SRR10099991 | PRJNA564423 | trinity transcriptome assembly and transdecoder |  |
| single cell transcriptome | NCBI SRA | Euglenida_sp_P3 | SRR10099982 | PRJNA564423 | trinity transcriptome assembly and transdecoder |  |
| single cell transcriptome | NCBI SRA | Euglenida_sp_P6 | SRR10099979 | PRJNA564423 | trinity transcriptome assembly and transdecoder |  |
| single cell transcriptome | NCBI SRA | Euglenida_sp_R5 | SRR10099974 | PRJNA564423 | trinity transcriptome assembly and transdecoder |  |
| single cell transcriptome | NCBI SRA | Euglenida_sp_R7 | SRR10099972 | PRJNA564423 | trinity transcriptome assembly and transdecoder |  |
| single cell transcriptome | NCBI SRA | Euglenida_sp_S1 | SRR10099970 | PRJNA564423 | trinity transcriptome assembly and transdecoder |  |
| single cell transcriptome | NCBI SRA | Euglenida_sp_X3 | SRR10100039 | PRJNA564423 | trinity transcriptome assembly and transdecoder |  |
| single cell transcriptome | NCBI SRA | Kinetoplastida_sp_10W | SRR10100019 | PRJNA564423 | trinity transcriptome assembly and transdecoder |  |
| single cell transcriptome | NCBI SRA | Kinetoplastida_sp_19W | SRR10099975 | PRJNA564423 | trinity transcriptome assembly and transdecoder |  |
| single cell transcriptome | NCBI SRA | Kinetoplastida_sp_23W | SRR10099964 | PRJNA564423 | trinity transcriptome assembly and transdecoder |  |
| single cell transcriptome | NCBI SRA | Kinetoplastida_sp_2W | SRR10100069 | PRJNA564423 | trinity transcriptome assembly and transdecoder |  |
| single cell transcriptome | NCBI SRA | Kinetoplastida_sp_7W | SRR10100051 | PRJNA564423 | trinity transcriptome assembly and transdecoder |  |
| single cell transcriptome | NCBI SRA | Kinetoplastida_sp_9W | SRR10100030 | PRJNA564423 | trinity transcriptome assembly and transdecoder |  |
| single cell transcriptome | NCBI SRA | Kinetoplastida_sp_A9 | SRR10100032 | PRJNA564423 | trinity transcriptome assembly and transdecoder |  |
| single cell transcriptome | NCBI SRA | Kinetoplastida_sp_C1 | SRR10100029 | PRJNA564423 | trinity transcriptome assembly and transdecoder |  |
| single cell transcriptome | NCBI SRA | Kinetoplastida_sp_C5 | SRR10100027 | PRJNA564423 | trinity transcriptome assembly and transdecoder |  |
| single cell transcriptome | NCBI SRA | Kinetoplastida_sp_C6 | SRR10100026 | PRJNA564423 | trinity transcriptome assembly and transdecoder |  |
| single cell transcriptome | NCBI SRA | Kinetoplastida_sp_E1 | SRR10100024 | PRJNA564423 | trinity transcriptome assembly and transdecoder |  |
| single cell transcriptome | NCBI SRA | Kinetoplastida_sp_L2 | SRR10100002 | PRJNA564423 | trinity transcriptome assembly and transdecoder |  |
| single cell transcriptome | NCBI SRA | Kinetoplastida_sp_L7 | SRR10099999 | PRJNA564423 | trinity transcriptome assembly and transdecoder |  |
| single cell transcriptome | NCBI SRA | Kinetoplastida_sp_N5 | SRR10099990 | PRJNA564423 | trinity transcriptome assembly and transdecoder |  |
| single cell transcriptome | NCBI SRA | Kinetoplastida_sp_X6 | SRR10100038 | PRJNA564423 | trinity transcriptome assembly and transdecoder |  |
| single cell transcriptome | NCBI SRA | Kinetoplastida_sp_Z3 | SRR10100062 | PRJNA564423 | trinity transcriptome assembly and transdecoder |  |

58. Harkins KM, Schwartz RS, Cartwright RA, Stone AC. Phylogenomic reconstruction supports supercontinent origins for *Leishmania*. Infect Genet Evol. 2016;38: 101–109. doi:10.1016/j.meegid.2015.11.030

59. Maruyama SR, de Santana AKM, Takamiya NT, Takahashi TY, Rogerio LA, Oliveira CAB, et al. Non-*Leishmania* Parasite in Fatal Visceral Leishmaniasis-Like Disease, Brazil. Emerg Infect Dis. 2019;25: 2088–2092. doi:10.3201/eid2511.181548

60. Nenarokova A, Záhonová K, Krasilnikova M, Gahura O, McCulloch R, Zíková A, et al. Causes and Effects of Loss of Classical Nonhomologous End Joining Pathway in Parasitic Eukaryotes. mBio. 2019;10: e01541-19. doi:10.1128/mBio.01541-19

61. Gawryluk RMR, Del Campo J, Okamoto N, Strassert JFH, Lukeš J, Richards TA, et al. Morphological Identification and Single-Cell Genomics of Marine Diplonemids. Curr Biol. 2016;26: 3053–3059. doi:10.1016/j.cub.2016.09.013

62. Wideman JG, Monier A, Rodríguez-Martínez R, Leonard G, Cook E, Poirier C, et al. Unexpected mitochondrial genome diversity revealed by targeted single-cell genomics of heterotrophic flagellated protists. Nat Microbiol. 2020;5: 154–165. doi:10.1038/s41564-019-0605-4

63. Wideman JG, Lax G, Leonard G, Milner DS, Rodríguez-Martínez R, Simpson AGB, et al. A single-cell genome reveals diplonemid-like ancestry of kinetoplastid mitochondrial gene structure. Philosophical Transactions of the Royal Society B: Biological Sciences. 2019;374: 20190100. doi:10.1098/rstb.2019.0100

64. Zakharova A, Saura A, Butenko A, Podešvová L, Warmusová S, Kostygov AYu, et al. A New Model Trypanosomatid, *Novymonas esmeraldas*: Genomic Perception of Its “Candidatus *Pandoraea novymonadis*” Endosymbiont. mBio. 12: e01606-21. doi:10.1128/mBio.01606-21

65. Kořený L, Sobotka R, Kovářová J, Gnipová A, Flegontov P, Horváth A, et al. Aerobic kinetoplastid flagellate *Phytomonas* does not require heme for viability. Proc Natl Acad Sci U S A. 2012;109: 3808–3813. doi:10.1073/pnas.1201089109

66. Alves JM, Klein CC, da Silva FM, Costa-Martins AG, Serrano MG, Buck GA, et al. Endosymbiosis in trypanosomatids: the genomic cooperation between bacterium and host in the synthesis of essential amino acids is heavily influenced by multiple horizontal gene transfers. BMC Evol Biol. 2013;13: 190. doi:10.1186/1471-2148-13-190

67. Gerasimov E, Zemp N, Schmid-Hempel R, Schmid-Hempel P, Yurchenko V. Genomic Variation among Strains of *Crithidia bombi* and *C. expoeki*. mSphere. 2019;4: e00482-19. doi:10.1128/mSphere.00482-19

68. Runckel C, DeRisi J, Flenniken ML. A Draft Genome of the Honey Bee Trypanosomatid Parasite *Crithidia mellificae*. PLoS ONE. 2014;9: e95057. doi:10.1371/journal.pone.0095057

69. Gupta AK, Srivastava S, Singh A, Singh S. De Novo Whole-Genome Sequence and Annotation of a *Leishmania* Strain Isolated from a Case of Post-Kala-Azar Dermal Leishmaniasis. Genome Announc. 2015;3: e00809-15. doi:10.1128/genomeA.00809-15

70. Batra D, Lin W, Rowe LA, Sheth M, Zheng Y, Loparev V, et al. Draft Genome Sequence of French Guiana *Leishmania (Viannia) guyanensis* Strain 204-365, Assembled Using Long Reads. Microbiol Resour Announc. 2018;7: e01421-18. doi:10.1128/MRA.01421-18

71. Lin W, Batra D, Narayanan V, Rowe LA, Sheth M, Zheng Y, et al. First Draft Genome Sequence of *Leishmania (Viannia) lainsoni* Strain 216-34, Isolated from a Peruvian Clinical Case. Microbiol Resour Announc. 2019;8: e01524-18. doi:10.1128/MRA.01524-18

72. Warren WC, Akopyants NS, Dobson DE, Hertz-Fowler C, Lye L-F, Myler PJ, et al. Genome Assemblies across the Diverse Evolutionary Spectrum of *Leishmania* Protozoan Parasites. Microbiol Resour Announc. 10: e00545-21. doi:10.1128/MRA.00545-21

73. Reis-Cunha JL, Baptista RP, Rodrigues-Luiz GF, Coqueiro-Dos-Santos A, Valdivia HO, de Almeida LV, et al. Whole genome sequencing of *Trypanosoma cruzi* field isolates reveals extensive genomic variability and complex aneuploidy patterns within TcII DTU. BMC Genomics. 2018;19: 816. doi:10.1186/s12864-018-5198-4

74. Callejas-Hernández F, Rastrojo A, Poveda C, Gironès N, Fresno M. Genomic assemblies of newly sequenced *Trypanosoma cruzi* strains reveal new genomic expansion and greater complexity. Sci Rep. 2018;8: 14631. doi:10.1038/s41598-018-32877-2

75. Baptista RP, Reis-Cunha JL, DeBarry JD, Chiari E, Kissinger JC, Bartholomeu DC, et al. Assembly of highly repetitive genomes using short reads: the genome of discrete typing unit III *Trypanosoma cruzi* strain 231. Microb Genom. 2018;4. doi:10.1099/mgen.0.000156

76. Reis-Cunha JL, Rodrigues-Luiz GF, Valdivia HO, Baptista RP, Mendes TAO, de Morais GL, et al. Chromosomal copy number variation reveals differential levels of genomic plasticity in distinct *Trypanosoma cruzi* strains. BMC Genomics. 2015;16: 499. doi:10.1186/s12864-015-1680-4

77. Callejas-Hernández F, Gironès N, Fresno M. Genome Sequence of *Trypanosoma cruzi* Strain Bug2148. Genome Announc. 2018;6: e01497-17. doi:10.1128/genomeA.01497-17

78. Hamilton PB, Lewis MD, Cruickshank C, Gaunt MW, Yeo M, Llewellyn MS, et al. Identification and lineage genotyping of South American trypanosomes using fluorescent fragment length barcoding. Infect Genet Evol. 2011;11: 44–51. doi:10.1016/j.meegid.2010.10.012

79. Singh N, Chikara S, Sundar S. SOLiD^TM^ Sequencing of Genomes of Clinical Isolates of *Leishmania donovani* from India Confirm *Leptomonas* Co-Infection and Raise Some Key Questions. PLOS ONE. 2013;8: e55738. doi:10.1371/journal.pone.0055738

80. Tikhonenkov DV, Gawryluk RMR, Mylnikov AP, Keeling PJ. First finding of free-living representatives of Prokinetoplastina and their nuclear and mitochondrial genomes. Sci Rep. 2021;11: 2946. doi:10.1038/s41598-021-82369-z

81. Yazaki E, Ishikawa SA, Kume K, Kumagai A, Kamaishi T, Tanifuji G, et al. Global Kinetoplastea phylogeny inferred from a large-scale multigene alignment including parasitic species for better understanding transitions from a free-living to a parasitic lifestyle. Genes Genet Syst. 2017;92: 35–42. doi:10.1266/ggs.16-00056

82. Kaur B, Záhonová K, Valach M, Faktorová D, Prokopchuk G, Burger G, et al. Gene fragmentation and RNA editing without borders: eccentric mitochondrial genomes of diplonemids. Nucleic Acids Res. 2020;48: 2694–2708. doi:10.1093/nar/gkz1215

83. Valach M, Moreira S, Hoffmann S, Stadler PF, Burger G. Keeping it complicated: Mitochondrial genome plasticity across diplonemids. Sci Rep. 2017;7: 14166. doi:10.1038/s41598-017-14286-z

84. Hasni I, Chelkha N, Baptiste E, Mameri MR, Lachuer J, Plasson F, et al. Investigation of potential pathogenicity of *Willaertia magna* by investigating the transfer of bacteria pathogenicity genes into its genome. Sci Rep. 2019;9: 18318. doi:10.1038/s41598-019-54580-6

85. Butler CE, Jaskowska E, Kelly S. Genome Sequence of *Phytomonas françai*, a Cassava (Manihot esculenta) Latex Parasite. Genome Announc. 2017;5: e01266-16. doi:10.1128/genomeA.01266-16

86. Opperdoes FR, Butenko A, Zakharova A, Gerasimov ES, Zimmer SL, Lukeš J, et al. The Remarkable Metabolism of *Vickermania ingenoplastis*: Genomic Predictions. Pathogens. 2021;10: 68. doi:10.3390/pathogens10010068

87. Manna PT, Kelly S, Field MC. Adaptin evolution in kinetoplastids and emergence of the variant surface glycoprotein coat in African trypanosomatids. Mol Phylogenet Evol. 2013;67: 123–128. doi:10.1016/j.ympev.2013.01.002

88. Albanaz ATS, Gerasimov ES, Shaw JJ, Sádlová J, Lukeš J, Volf P, et al. Genome Analysis of *Endotrypanum* and *Porcisia* spp., Closest Phylogenetic Relatives of *Leishmania*, Highlights the Role of Amastins in Shaping Pathogenicity. Genes. 2021;12: 444. doi:10.3390/genes12030444

89. Ishemgulova A, Butenko A, Kortišová L, Boucinha C, Grybchuk-Ieremenko A, Morelli KA, et al. Molecular mechanisms of thermal resistance of the insect trypanosomatid *Crithidia thermophila*. PLOS ONE. 2017;12: e0174165. doi:10.1371/journal.pone.0174165

90. Leger MM, Eme L, Hug LA, Roger AJ. Novel Hydrogenosomes in the Microaerophilic Jakobid *Stygiella incarcerata*. Mol Biol Evol. 2016;33: 2318–2336. doi:10.1093/molbev/msw103

91. Horváthová L, Žárský V, Pánek T, Derelle R, Pyrih J, Motyčková A, et al. Analysis of diverse eukaryotes suggests the existence of an ancestral mitochondrial apparatus derived from the bacterial type II secretion system. Nat Commun. 2021;12: 2947. doi:10.1038/s41467-021-23046-7

92. Soukal P, Hrdá Š, Karnkowska A, Milanowski R, Szabová J, Hradilová M, et al. Heterotrophic euglenid *Rhabdomonas costata* resembles its phototrophic relatives in many aspects of molecular and cell biology. Sci Rep. 2021;11: 13070. doi:10.1038/s41598-021-92174-3

93. Carrington M, Dóró E, Forlenza M, Wiegertjes GF, Kelly S. Transcriptome Sequence of the Bloodstream Form of *Trypanoplasma borreli*, a Hematozoic Parasite of Fish Transmitted by Leeches. Genome Announc. 2017;5: e01712-16. doi:10.1128/genomeA.01712-16

94. Yoshida Y, Tomiyama T, Maruta T, Tomita M, Ishikawa T, Arakawa K. De novo assembly and comparative transcriptome analysis of *Euglena gracilis* in response to anaerobic conditions. BMC Genomics. 2016;17: 182. doi:10.1186/s12864-016-2540-6

95. Záhonová K, Füssy Z, Birčák E, Novák Vanclová AMG, Klimeš V, Vesteg M, et al. Peculiar features of the plastids of the colourless alga *Euglena longa* and photosynthetic euglenophytes unveiled by transcriptome analyses. Sci Rep. 2018;8: 17012. doi:10.1038/s41598-018-35389-1

96. Harding T, Brown MW, Simpson AGB, Roger AJ. Osmoadaptative Strategy and Its Molecular Signature in Obligately Halophilic Heterotrophic Protists. Genome Biol Evol. 2016;8: 2241–2258. doi:10.1093/gbe/evw152

97. Keeling PJ, Burki F, Wilcox HM, Allam B, Allen EE, Amaral-Zettler LA, et al. The Marine Microbial Eukaryote Transcriptome Sequencing Project (MMETSP): Illuminating the Functional Diversity of Eukaryotic Life in the Oceans through Transcriptome Sequencing. PLOS Biology. 2014;12: e1001889. doi:10.1371/journal.pbio.1001889

98. Ebenezer TE, Zoltner M, Burrell A, Nenarokova A, Novák Vanclová AMG, Prasad B, et al. Transcriptome, proteome and draft genome of *Euglena gracilis*. BMC Biology. 2019;17: 11. doi:10.1186/s12915-019-0626-8

99. Jackson AP, Otto TD, Aslett M, Armstrong SD, Bringaud F, Schlacht A, et al. Kinetoplastid Phylogenomics Reveals the Evolutionary Innovations Associated with the Origins of Parasitism. Curr Biol. 2016;26: 161–172. doi:10.1016/j.cub.2015.11.055

100. David V, Flegontov P, Gerasimov E, Tanifuji G, Hashimi H, Logacheva MD, et al. Gene Loss and Error-Prone RNA Editing in the Mitochondrion of *Perkinsela*, an Endosymbiotic Kinetoplastid. mBio. 2015;6: e01498-01415. doi:10.1128/mBio.01498-15

101. Liechti N, Schürch N, Bruggmann R, Wittwer M. Nanopore sequencing improves the draft genome of the human pathogenic amoeba *Naegleria fowleri*. Sci Rep. 2019;9: 16040. doi:10.1038/s41598-019-52572-0

102. Fritz-Laylin LK, Prochnik SE, Ginger ML, Dacks JB, Carpenter ML, Field MC, et al. The genome of *Naegleria gruberi* illuminates early eukaryotic versatility. Cell. 2010;140: 631–642. doi:10.1016/j.cell.2010.01.032

103. Gray MW, Burger G, Derelle R, Klimeš V, Leger MM, Sarrasin M, et al. The draft nuclear genome sequence and predicted mitochondrial proteome of *Andalucia godoyi*, a protist with the most gene-rich and bacteria-like mitochondrial genome. BMC Biology. 2020;18: 22. doi:10.1186/s12915-020-0741-6

104. Liechti N, Schürch N, Bruggmann R, Wittwer M. The genome of *Naegleria lovaniensis*, the basis for a comparative approach to unravel pathogenicity factors of the human pathogenic amoeba *N. fowleri*. BMC Genomics. 2018;19: 654. doi:10.1186/s12864-018-4994-1

105. Bradwell KR, Koparde VN, Matveyev AV, Serrano MG, Alves JMP, Parikh H, et al. Genomic comparison of *Trypanosoma conorhini* and *Trypanosoma rangeli* to *Trypanosoma cruzi* strains of high and low virulence. BMC Genomics. 2018;19: 770. doi:10.1186/s12864-018-5112-0

106. Porcel BM, Denoeud F, Opperdoes F, Noel B, Madoui M-A, Hammarton TC, et al. The Streamlined Genome of *Phytomonas* spp. Relative to Human Pathogenic Kinetoplastids Reveals a Parasite Tailored for Plants. PLOS Genetics. 2014;10: e1004007. doi:10.1371/journal.pgen.1004007

107. Hébert L, Moumen B, Madeline A, Steinbiss S, Lakhdar L, Van Reet N, et al. First Draft Genome Sequence of the Dourine Causative Agent: *Trypanosoma equiperdum* Strain OVI. J Genomics. 2017;5: 1–3. doi:10.7150/jgen.17904

108. Almutairi H, Urbaniak MD, Bates MD, Jariyapan N, Al-Salem WS, Dillon RJ, et al. Chromosome-Scale Assembly of the Complete Genome Sequence of *Leishmania (Mundinia) orientalis*, Isolate LSCM4, Strain LV768. Microbiology Resource Announcements. 10: e00574-21. doi:10.1128/MRA.00574-21

109. Almutairi H, Urbaniak MD, Bates MD, Jariyapan N, Al-Salem WS, Dillon RJ, et al. Chromosome-Scale Assembly of the Complete Genome Sequence of *Leishmania (Mundinia) martiniquensis*, Isolate LSCM1, Strain LV760. Microbiol Resour Announc. 2021;10: e0005821. doi:10.1128/MRA.00058-21

110. Kelly S, Ivens A, Mott GA, O’Neill E, Emms D, Macleod O, et al. An Alternative Strategy for Trypanosome Survival in the Mammalian Bloodstream Revealed through Genome and Transcriptome Analysis of the Ubiquitous Bovine Parasite *Trypanosoma (Megatrypanum) theileri*. Genome Biol Evol. 2017;9: 2093–2109. doi:10.1093/gbe/evx152

111. Mott GA, Wilson R, Fernando A, Robinson A, MacGregor P, Kennedy D, et al. Targeting cattle-borne zoonoses and cattle pathogens using a novel trypanosomatid-based delivery system. PLoS Pathog. 2011;7: e1002340. doi:10.1371/journal.ppat.1002340

112. Stoco PH, Wagner G, Talavera-Lopez C, Gerber A, Zaha A, Thompson CE, et al. Genome of the avirulent human-infective trypanosome--*Trypanosoma rangeli*. PLoS Negl Trop Dis. 2014;8: e3176. doi:10.1371/journal.pntd.0003176

113. Carnes J, Anupama A, Balmer O, Jackson A, Lewis M, Brown R, et al. Genome and phylogenetic analyses of *Trypanosoma evansi* reveal extensive similarity to T. brucei and multiple independent origins for dyskinetoplasty. PLoS Negl Trop Dis. 2015;9: e3404. doi:10.1371/journal.pntd.0003404

114. Wang W, Peng D, Baptista RP, Li Y, Kissinger JC, Tarleton RL. Strain-specific genome evolution in *Trypanosoma cruzi*, the agent of Chagas disease. PLoS Pathog. 2021;17: e1009254. doi:10.1371/journal.ppat.1009254

115. Berná L, Rodriguez M, Chiribao ML, Parodi-Talice A, Pita S, Rijo G, et al. Expanding an expanded genome: long-read sequencing of *Trypanosoma cruzi*. Microb Genom. 2018;4. doi:10.1099/mgen.0.000177

116. Franzén O, Ochaya S, Sherwood E, Lewis MD, Llewellyn MS, Miles MA, et al. Shotgun sequencing analysis of *Trypanosoma cruzi* I Sylvio X10/1 and comparison with *T. cruzi* VI CL Brener. PLoS Negl Trop Dis. 2011;5: e984. doi:10.1371/journal.pntd.0000984

117. Franzén O, Talavera-López C, Ochaya S, Butler CE, Messenger LA, Lewis MD, et al. Comparative genomic analysis of human infective *Trypanosoma cruzi* lineages with the bat-restricted subspecies *T. cruzi* *marinkellei*. BMC Genomics. 2012;13: 531. doi:10.1186/1471-2164-13-531

118. Contreras VT, Araujo-Jorge TC, Bonaldo MC, Thomaz N, Barbosa HS, Meirelles M de N, et al. Biological aspects of the Dm 28c clone of *Trypanosoma cruzi* after metacyclogenesis in chemically defined media. Mem Inst Oswaldo Cruz. 1988;83: 123–133. doi:10.1590/s0074-02761988000100016

119. Grisard EC, Teixeira SMR, de Almeida LGP, Stoco PH, Gerber AL, Talavera-López C, et al. *Trypanosoma cruzi* Clone Dm28c Draft Genome Sequence. Genome Announc. 2014;2: e01114-13. doi:10.1128/genomeA.01114-13

120. Weatherly DB, Boehlke C, Tarleton RL. Chromosome level assembly of the hybrid *Trypanosoma cruzi* genome. BMC Genomics. 2009;10: 255. doi:10.1186/1471-2164-10-255

121. El-Sayed NM, Myler PJ, Bartholomeu DC, Nilsson D, Aggarwal G, Tran A-N, et al. The genome sequence of *Trypanosoma cruzi*, etiologic agent of Chagas disease. Science. 2005;309: 409–415. doi:10.1126/science.1112631

122. Abbas AH, Silva Pereira S, D’Archivio S, Wickstead B, Morrison LJ, Hall N, et al. The Structure of a Conserved Telomeric Region Associated with Variant Antigen Loci in the Blood Parasite *Trypanosoma congolense*. Genome Biol Evol. 2018;10: 2458–2473. doi:10.1093/gbe/evy186

123. Jackson AP, Berry A, Aslett M, Allison HC, Burton P, Vavrova-Anderson J, et al. Antigenic diversity is generated by distinct evolutionary mechanisms in African trypanosome species. Proc Natl Acad Sci U S A. 2012;109: 3416–3421. doi:10.1073/pnas.1117313109

124. Berriman M, Ghedin E, Hertz-Fowler C, Blandin G, Renauld H, Bartholomeu DC, et al. The genome of the African trypanosome *Trypanosoma brucei*. Science. 2005;309: 416–22. doi:10.1126/science.1112642

125. Müller LSM, Cosentino RO, Förstner KU, Guizetti J, Wedel C, Kaplan N, et al. Genome organization and DNA accessibility control antigenic variation in trypanosomes. Nature. 2018;563: 121. doi:10.1038/s41586-018-0619-8

126. Jackson AP, Sanders M, Berry A, McQuillan J, Aslett MA, Quail MA, et al. The genome sequence of *Trypanosoma brucei gambiense*, causative agent of chronic human african trypanosomiasis. PLoS Negl Trop Dis. 2010;4: e658. doi:10.1371/journal.pntd.0000658

127. Skalický T, Dobáková E, Wheeler RJ, Tesařová M, Flegontov P, Jirsová D, et al. Extensive flagellar remodeling during the complex life cycle of *Paratrypanosoma*, an early-branching trypanosomatid. Proc Natl Acad Sci USA. 2017;114: 11757–11762. doi:10.1073/pnas.1712311114

128. Nasereddin A, Schweynoch C, Schonian G, Jaffe CL. Characterization of *Leishmania* *(Leishmania) tropica* axenic amastigotes. Acta Trop. 2010;113: 72–79. doi:10.1016/j.actatropica.2009.09.009

129. Raymond F, Boisvert S, Roy G, Ritt J-F, Légaré D, Isnard A, et al. Genome sequencing of the lizard parasite *Leishmania tarentolae* reveals loss of genes associated to the intracellular stage of human pathogenic species. Nucleic Acids Research. 2011 [cited 18 Oct 2011]. doi:10.1093/nar/gkr834

130. Kraeva N, Butenko A, Hlaváčová J, Kostygov A, Myškova J, Grybchuk D, et al. *Leptomonas seymouri*: Adaptations to the Dixenous Life Cycle Analyzed by Genome Sequencing, Transcriptome Profiling and Co-infection with *Leishmania donovani*. PLoS Pathog. 2015;11: e1005127. doi:10.1371/journal.ppat.1005127

131. Flegontov P, Butenko A, Firsov S, Kraeva N, Eliáš M, Field MC, et al. Genome of *Leptomonas pyrrhocoris*: a high-quality reference for monoxenous trypanosomatids and new insights into evolution of *Leishmania*. Sci Rep. 2016;6: 23704. doi:10.1038/srep23704

132. Llanes A, Restrepo CM, Del Vecchio G, Anguizola FJ, Lleonart R. The genome of *Leishmania* *panamensis*: insights into genomics of the *L. (Viannia)* subgenus. Sci Rep. 2015;5: 8550. doi:10.1038/srep08550

133. Rogers MB, Hilley JD, Dickens NJ, Wilkes J, Bates PA, Depledge DP, et al. Chromosome and gene copy number variation allow major structural change between species and strains of *Leishmania*. Genome Res. 2011;21: 2129–2142. doi:10.1101/gr.122945.111

134. González-de la Fuente S, Peiró-Pastor R, Rastrojo A, Moreno J, Carrasco-Ramiro F, Requena JM, et al. Resequencing of the *Leishmania infantum* (strain JPCM5) genome and de novo assembly into 36 contigs. Sci Rep. 2017;7: 18050. doi:10.1038/s41598-017-18374-y

135. Lypaczewski P, Hoshizaki J, Zhang W-W, McCall L-I, Torcivia-Rodriguez J, Simonyan V, et al. A complete *Leishmania donovani* reference genome identifies novel genetic variations associated with virulence. Sci Rep. 2018;8: 16549. doi:10.1038/s41598-018-34812-x

136. Zhang WW, Ramasamy G, McCall L-I, Haydock A, Ranasinghe S, Abeygunasekara P, et al. Genetic analysis of *Leishmania donovani* tropism using a naturally attenuated cutaneous strain. PLoS Pathog. 2014;10: e1004244. doi:10.1371/journal.ppat.1004244

137. Downing T, Imamura H, Decuypere S, Clark TG, Coombs GH, Cotton JA, et al. Whole genome sequencing of multiple *Leishmania donovani* clinical isolates provides insights into population structure and mechanisms of drug resistance. Genome Res. 2011;21: 2143–2156. doi:10.1101/gr.123430.111

138. Peacock CS, Seeger K, Harris D, Murphy L, Ruiz JC, Quail MA, et al. Comparative genomic analysis of three *Leishmania* species that cause diverse human disease. Nat Genet. 2007;39: 839–847. doi:10.1038/ng2053

139. Real F, Vidal RO, Carazzolle MF, Mondego JMC, Costa GGL, Herai RH, et al. The Genome Sequence of *Leishmania (Leishmania) amazonensis*: Functional Annotation and Extended Analysis of Gene Models. DNA Res. 2013;20: 567–581. doi:10.1093/dnares/dst031

140. Midha S, Rigden DJ, Siozios S, Hurst GDD, Jackson AP. *Bodo saltans* (Kinetoplastida) is dependent on a novel *Paracaedibacter*-like endosymbiont that possesses multiple putative toxin-antitoxin systems. ISME J. 2021;15: 1680–1694. doi:10.1038/s41396-020-00879-6

141. Opperdoes FR, Butenko A, Flegontov P, Yurchenko V, Lukeš J. Comparative Metabolism of Free-living *Bodo saltans* and Parasitic Trypanosomatids. J Eukaryot Microbiol. 2016;63: 657–678. doi:10.1111/jeu.12315

142. Davey JW, Catta-Preta CMC, James S, Forrester S, Motta MCM, Ashton PD, et al. Chromosomal assembly of the nuclear genome of the endosymbiont-bearing trypanosomatid *Angomonas deanei*. G3 (Bethesda). 2021;11: jkaa018. doi:10.1093/g3journal/jkaa018

143. Lax G, Kolisko M, Eglit Y, Lee WJ, Yubuki N, Karnkowska A, et al. Multigene phylogenetics of euglenids based on single-cell transcriptomics of diverse phagotrophs. Molecular Phylogenetics and Evolution. 2021;159: 107088. doi:10.1016/j.ympev.2021.107088
